# Serum metabolomics identifies ganglioside dysregulation and pyruvate dehydrogenase complex–related metabolic disturbances in pediatric drug-resistant epilepsy

**DOI:** 10.3389/fped.2026.1952153

**Published:** 2026-09-29

**Authors:** Eman Y. Abu-Rish, Reem H. AlMalki, Amira Masri, Lina A. Dahabiyeh, Roaa Alnajjar, Rula M. Darwish, Anas M. Abdel Rahman

**Affiliations:** 1 Department of Biopharmaceutics and Clinical Pharmacy, School of Pharmacy, The University of Jordan, Amman, Jordan; 2 Metabolomics Section, Precision Medicine Laboratory Department, Genomics Medicine Center of Excellence, King Faisal Specialist Hospital and Research Centre (KFSHRC), Riyadh, Saudi Arabia; 3 Faculty of Medicine, The University of Jordan, Amman, Jordan; 4 Department of Pharmaceutical Sciences, School of Pharmacy, The University of Jordan, Amman, Jordan; 5 Department of Pharmaceutics and Pharmaceutical Technology, School of Pharmacy, The University of Jordan, Amman, Jordan; 6 Department of Biochemistry and Molecular Medicine, College of Medicine, Alfaisal University, Riyadh, Saudi Arabia

**Keywords:** children, metabolomics, mitochondrial dysfunction, refractory epilepsy, seizure, TCA cycle, thiamine diphosphate

## Abstract

**Introduction:**

Despite advances in epilepsy management, the exact causes of epilepsy remain unclear, and diagnostic biomarkers are still lacking. Besides, about one-third of cases involve drug-resistant epilepsy (DRE).

**Methods:**

This study employed untargeted metabolomics to identify potential metabolic signatures in the sera of pediatric patients with epilepsy using liquid chromatography-high-resolution mass spectrometry.

**Results:**

Our results revealed several disrupted energetic pathways and metabolites indicative of oxidative stress and mitochondrial dysfunction in both controlled epilepsy (CE) and DRE groups. Seventy-five metabolites were significantly elevated in CE compared to healthy controls (HC), including ethenoadenosine, 6-methyladenine, and fatty acyl-carnitine conjugates and intermediates. Additionally, 144 metabolites were decreased in CE relative to HC, including various fatty acyl-CoA intermediates, GM3 ganglioside, and amino acids. Key disrupted pathways in CE included pyrimidine and purine metabolism, phenylalanine, tyrosine, and tryptophan biosynthesis, pyruvate metabolism, and glycolysis/gluconeogenesis. In DRE, 25 metabolites were significantly reduced compared to non-DRE (NDRE), notably S-acetyldihydrolipoamide, 2-(alpha-hydroxyethyl)-thiamine diphosphate (HE-TDP), and S-adenosylmethionine (SAM). Metabolites involved in glycerophospholipid biosynthesis were decreased, along with lower levels of GM2 ganglioside. The most affected pathways in DRE included the tricarboxylic acid (TCA) cycle, pyruvate metabolism, glycolysis/gluconeogenesis, and cysteine and methionine metabolism.

**Conclusion:**

The observed alterations in S-acetyldihydrolipoamide, HE-TDP, and SAM support their further investigation as candidate monitoring biomarkers and as metabolites potentially involved in therapeutically relevant pathways in DRE. The alterations observed in HE-TDP, SAM, GM2, PS, and PE generate the hypothesis that pathways involving thiamine-dependent energy metabolism, methylation, ganglioside metabolism, and mitochondrial membrane function may be relevant to DRE. However, the present observational study did not evaluate supplementation, causality, efficacy, or safety. Consequently, any therapeutic application remains speculative and would require mechanistic validation, preclinical assessment, and appropriately designed clinical trials.

## Introduction

Epilepsy is a chronic neurological disorder affecting approximately 50 million people worldwide (1). It is characterized by recurrent, unprovoked seizures caused by factors such as genetic mutations, structural abnormalities, and metabolic or mitochondrial dysfunction (2). Mitochondrial dysfunction and oxidative stress play crucial roles in the pathology of epilepsy, lowering the seizure threshold and promoting neuronal hyperexcitability (3). Multiple pathways may contribute to this dysfunction (2). Prior research has highlighted the roles of the tricarboxylic acid (TCA) cycle and the pyruvate pathway in mitochondrial dysfunction and epilepsy development (3–5). However, key aspects of the disease process and potential treatment options remain unclear.

Several reports have used metabolomics to explore the pathogenesis of epilepsy and drug-resistant epilepsy (DRE) (6, 7). Alterations in amino acids and organic acids, such as glutamate, lactate, and citrate, have been identified in patients with epilepsy, along with disruptions in the metabolic pathways of alanine, aspartate, glutamate, glycine, serine, and threonine; glycerophospholipid metabolism; and glyoxylate and dicarboxylate metabolism (6). However, the underlying mechanisms of epilepsy remain unclear, and specific biomarkers are still lacking. Multi-omics studies indicate that DRE pathophysiology involves changes in genes encoding neurotransmitter receptors, drug transporters, ion channels, and drug targets, as well as alterations in metabolite levels related to neurotransmitter signaling, energy pathways, oxidative stress, and neuroinflammation (7). Nonetheless, few human studies have compared the metabolomes of patients with DRE to those without DRE, and those that have either show no clear differences (6, 8, 9) or provide inconsistent findings (4, 7, 10, 11). Additionally, most studies analyzing the metabolome in patients with epilepsy have used either proton nuclear magnetic resonance (1H NMR) (6, 8–10) or gas chromatography-mass spectrometry (GC-MS) (11–13), with only a few reports employing liquid chromatography-mass spectrometry (LC-MS) analysis (4, 7, 14).

This study aims to explore the network linking mitochondrial dysfunction, oxidative stress, and energy pathways that may underlie the pathological process in epilepsy. Understanding this network may help develop new diagnostic methods for epilepsy, which are especially needed because current diagnosis primarily depends on ictal electroencephalogram (EEG) results and physician assessments of symptoms (6). Additionally, about one-third of patients with epilepsy are resistant to existing anti-seizure medications (ASMs), despite the availability of over 20 different ASMs (15, 16). Therefore, there is an urgent need to identify new therapeutic targets to improve treatment for resistant cases. Furthermore, most human studies have been conducted in adults, with fewer reports in pediatric epilepsy (4, 5, 8).

Therefore, in this study, we present untargeted LC-MS-based metabolomics profiling of sera from pediatric patients with epilepsy and healthy controls (HC). The metabolome of patients with controlled epilepsy (CE) was compared to HC, and those with DRE were compared to those with non-DRE (NDRE). Therefore, this study's findings will expand our understanding of the pathophysiology of pediatric epilepsy and DRE and could provide insights into new diagnostic biomarkers and therapeutic targets for epilepsy and DRE.

## Methods

### Study subjects and study protocol

A total of 150 pediatric patients with epilepsy under the age of 18 presented to the neurology clinic at the University of Jordan Hospital between June 2020 and June 2022. Patients with other neurological conditions were excluded, as were those with conditions that might affect inflammatory status, including active infection, thyroid dysfunction, chronic inflammatory diseases, autoimmune diseases, cancer, renal impairment, hepatic impairment, surgery or major trauma in the previous month, or use of immunosuppressive or immunomodulatory medications in the six weeks prior to the study.

Patients were divided into two main groups based on epilepsy control status. Those whose seizures were controlled with ASMs and who were seizure-free for at least 3 months were considered the CE group (*n* = 54). The uncontrolled epilepsy (UCE) group (*n* = 96) included two subgroups based on response to ASMs: patients with DRE (*n* = 25) (uncontrolled and drug-resistant) and those with NDRE (*n* = 71) (uncontrolled but non-drug-resistant). Drug-resistant epilepsy was defined by the International League Against Epilepsy as the failure of at least two ASMs used at maximum therapeutic doses to control seizures (17). Those with NDRE included patients whose epilepsy remains uncontrolled while using only a trial of one ASM or two ASMs at doses before reaching the maximum tolerated therapeutic doses, thus have not yet met the criteria for drug resistance and are still uncontrolled at that level of treatment (NDRE = UCE - DRE). Additionally, the HC group of age- and sex-matched children (*n* = 15) was included in this study. The small sample size of this group is justified by the difficulty of obtaining consent to provide a blood sample from healthy children unless they had routine blood testing.

Patient demographics and medical history were obtained from electronic medical records. This included age, gender, weight, height, Body Mass Index (BMI), age at epilepsy onset, duration of epilepsy, time since the last seizure, type of epilepsy (focal or generalized), magnetic resonance imaging (MRI) findings if available, number of anti-seizure medications used, and presence of developmental delay. Seizure severity was measured using the 7-item National Hospital Seizure Severity Scale (NHS3) (18). Previous studies have used this scale in pediatric epilepsy patients (19). In this study, we applied this scale to children aged 6 years and older, as some items, such as assessing urinary incontinence during a seizure, were not applicable to children younger than 6. The score was determined for all seizure types experienced by each patient, and the highest score was recorded. Data collection also involved consultation with parents/caregivers and the pediatric neurologist. Privacy rights of human subjects were observed, and informed consent was obtained from all parents/caregivers. This research adhered to the principles outlined in the Declaration of Helsinki, and all procedures were performed in compliance with relevant laws and institutional guidelines and were approved by the Ethics Review Board at the University of Jordan Hospital (IRB no. 328/2019).

### Sample collection, processing, and storage

Blood samples (3 mL) were collected from fasted children during routine lab tests, with written consent, in accordance with our standard protocol. The silica-gel serum separator tubes were left at room temperature for 30 min to clot, then centrifuged at 3,000 rpm for 10 min at 4 °C. Sera were aliquoted and immediately stored at −80 °C.

### Metabolites extraction

Metabolites were extracted following our lab standard protocol (20, 21). In detail, from a 50 µL serum sample, 345 µL of cold, absolute LC-MS methanol and 172.5 µL of chloroform were added. After vortexing for 30 s, 88 µL of LC-MS water was added, and the mixture was vortexed for another 30 s. Finally, 172.5 µL of chloroform and 172.5 µL of water were added, and the mixture was vortexed for 30 s before centrifugation for 5 min at 10,000 rpm at 4 °C (20). The upper aqueous phase of the extract was dried using a vacuum concentrator (Eppendorf, Germany). Before LC-MS analysis, dried samples were reconstituted in 100 µL of 50% mobile phase A: B [A: 0.1% formic acid in dH_2_O, B: 0.1% formic acid in (1:1 v/v) Acetonitrile (ACN): Methanol (MeOH)].

### LC-MS metabolomics

Metabolic profiles were analyzed using the Waters Acquity UPLC system coupled to a Xevo G2-S QTOF mass spectrometer equipped with an electrospray ionization (ESI) source (21). Metabolites were chromatographed on an ACQUITY UPLC XSelect HSS T3 column (100 × 2.1 mm, 2.5 μm, Waters Ltd., Elstree, UK). The mobile phase consisted of 0.1% formic acid in dH2O (solvent A) and 0.1% formic acid in 50% ACN: MeOH (solvent B). A gradient elution schedule was run as follows: 0–16 min, 95%–5% A; 16–19 min, 5% A; 19–20 min, 5%–95% A; 20–22 min, 5%–95% A, at a flow rate of 300 µL/min. MS spectra were acquired in positive and negative electrospray ionization (ESI+) and electrospray ionization (ESI-) modes. MS conditions were as follows: source temperature 150 °C, desolvation temperature 500 °C, capillary voltage 3.20 kV (ESI+) or 3 kV (ESI−), cone voltage 40 V, desolvation gas flow 800.0 L/h, and cone gas flow 50 L/h. In MSE mode, low and high collision energy functions were set to off and 10–50 V, respectively. The mass spectrometer was calibrated using sodium formate over the 100–1,200 Da range. Data were collected in continuum mode with the Masslynx™ V4.1 workstation (Waters Inc., Milford, Massachusetts, USA). Pooled QC samples were analyzed periodically throughout the analytical sequence to monitor instrument stability, signal drift, and analytical reproducibility. Features detected in the pooled QC samples with an RSD ≤30% were retained for subsequent analysis. Potential batch effects were assessed by examining QC-sample clustering and temporal signal trends across the injection sequence (22).

### Data processing, statistical analysis, and metabolite identification

The MS raw data were processed using a standard pipeline in Progenesis QI v.3.0 from Waters (Waters Technologies, Milford, MA, USA), including alignment based on m/z and ion signal retention time, peak picking, and signal filtering based on peak quality. Metabolite identification confidence was assigned according to the Metabolomics Standards Initiative (MSI) guidelines. Metabolites were annotated based on accurate precursor mass, isotopic distribution, and MS/MS fragmentation patterns by comparison with the Human Metabolome Database (HMDB), METLIN MS/MS library (23), and an in-house spectral library. A mass tolerance of 5 ppm was applied for mass matching. Eight metabolites were confirmed using authentic reference standards analyzed under matched analytical conditions and were classified as MSI level 1. The remaining metabolites, for which authentic-standard confirmation was not available, were classified as putatively annotated metabolites (MSI level 2). Exogenous compounds, such as drugs, food additives, and environmental compounds, were excluded from the final list.

Statistical analysis was performed using MetaboAnalyst version 5.0 (McGill University, Montreal, Canada) (http://www.metaboanalyst.ca), accessed on March 12, 2024 (24). Data sets (compounds and their abundances across different groups) were median-normalized, Pareto-scaled, and log-transformed to maintain normality. The normalized datasets were used to build orthogonal partial least squares-discriminant analysis (OPLS-DA) models. OPLS-DA models were evaluated using the model fitness (R2Y) and predictive ability (Q2) values with 100-fold permutation validation (25).

A univariate analysis was performed using Mass Profiler Professional software v.15.0 (Agilent, Santa Clara, CA, USA). A volcano plot was used to identify significantly altered mass features based on an unpaired t-test with FDR-adjusted *p* < 0.05 and a fold change (FC) cutoff of 2 (26). In accordance with the Pearson similarity test, a heatmap of changed components was generated using the Pearson distance measure. Pathway analysis associated with epilepsy was performed using MetaboAnalyst v5.0. For each between-group metabolite comparison, the fold change and its 95% confidence interval were calculated to describe the magnitude, direction, and precision of the observed difference. Effect-size estimates were interpreted alongside FDR-adjusted *P* values rather than using statistical significance alone to infer biological relevanceStatistical analyses of demographic and clinical data were conducted using SPSS version 27.0.1 (SPSS Inc., Chicago, IL). Categorical variables were presented as percentages with frequencies, and continuous variables were presented as mean ± standard deviation (SD). Normality of continuous variables was assessed using the Kolmogorov–Smirnov and Shapiro–Wilk tests. Pearson's chi-square test was used to assess associations between categorical variables. The Mann–Whitney U test or Kruskal–Wallis test was used to assess between-group differences. A *P*-value of < 0.05 was considered statistically significant. Effect size was calculated using the formula r = z/√N for the Mann–Whitney U test, where z is the standardized test statistic, and N is the number of observations. For the chi-square test, the formula ω=√(χ2/N)was used, where χ2 is Pearson's chi-square value, and N is the number of observations. The thresholds for r and *ω* are 0.1, 0.3, and 0.5 for small, medium, and large effects, respectively. For Kruskal–Wallis test effect size was calculated using two formulas, first *Eta*^2^ is calculated using *Eta*^2^= (H-k + 1)/ (n-k) where H is Kruskal–Wallis test, k is the number of groups and *n* is the number of observations then *Eta*^2^ is transformed to *f* using the formula *f* = √[*Eta*^2^/(1-*Eta*^2^)]. The thresholds for *f* are 0.1, 0.25, and 0.4 for small, medium, and large effects, respectively.

## Results

### Demographic and clinical characteristics of the study participants

The demographic characteristics of the study participants and the clinical features of patients with epilepsy are shown in Tables 1, 2, respectively. No significant demographic differences were found between the groups in any comparison (Table 1). As shown in Table 2, the mean age at seizure onset was similar across all epilepsy subgroups (*P*-value = 0.512). The duration since the last seizure was significantly the longest in the CE group (*P*-value = 0.000) which complies with the definition of each group. Seizure severity score was highest in the DRE group though not statistically significant (*P*-value = 0.280). The proportions of patients with focal and generalized epilepsy were comparable across all subgroups (*P*-value = 0.656). The DRE group had the highest proportions of patients with abnormal MRI results, those on combination therapy, and developmental delays, while the CE group had the lowest (*P*-value = 0.101, <0.001, 0.003, respectively).

**Table 1:** Demographic data of healthy controls and epilepsy patients.

Parameter	HC (*n* = 15)	CE (*n* = 54)	*P-*value*(effect size)^d^	DRE (*n* = 25)	NDRE (*n* = 71)	*P-*value* (effect size)^d^
Age (years)^a^^,^^b^	8.80 ± 3.76	10.35 ± 4.94	0.19 (0.158)	9.28 ± 5.06	7.68 ± 4.98	0.161 (0.143)
Weight (kg)^a^^,^^b^	31.85 ± 4.96	38.24 ± 22.05	0.312 (0.124)	34.26 ± 19.16	31.07 ± 20.94	0.488 (0.071)
Height (cm)^a^^,^^b^	129.65 ± 3.63	128.20 ± 38.66	0.589 (0.067)	123.29 ± 26.19	110.62 ± 43.81	0.343 (0.099)
BMI (kg/m2)^a^^,^^b^	18.14 ± 5.03	19.80 ± 5.38	0.225 (0.152)	21.38 ± 6.90	19.11 ± 6.07	0.233 (0.128)
Gender^c^						
Male *n* (%)	7 (46.67)	30 (55.56)	0.541	16 (64)	40 (56.34)	0.504 (0.068)
Female *n* (%)	8 (53.33)	24 (44.44)	(0.074)	9 (36)	31 (43.66)	

BMI, body mass index; CE, controlled epilepsy; DRE, drug-resistant epilepsy; HC, healthy control; NDRE, non-drug-resistant epilepsy; SD, standard deviation.

a Data are presented as mean ± SD.

b Mann–Whitney u test.

c Pearson Chi-square test. *Significant at *P*-value <0.05.

d effect size: r for Mann–Whitney U test and *ω* for chi-square test, thresholds are 0.1, 0.3 and 0.5 for small, medium and large effects, respectively.

**Table 2:** Clinical features of the epilepsy patients in the three subgroups.

Parameter	CE (*n* = 54)	DRE (*n* = 25)	NDRE (*n* = 71)	*P*-value*(effect size)^f^
Epilepsy at an early age (years)^a^^,^^b^	5.79 ± 4.76	4.35 ± 4.31	5.05 ± 4.56	0.512 (0.0.067)
Duration of epilepsy (years)^a^^,^^b^	4.31 ± 3.90	4.91 ± 4.11	2.26 ± 2.61	<0.001 (0.37)
Duration of time since last seizure (days)^a^^,^^b^	606.64 ± 1,041.69	29.42 ± 42.93	21.76 ± 49.61	0.000 (1.137)
Seizure severity (NHS3 score)^a^^,^^b^^,^^c^	8.86 ± 4.30	11.39 ± 6.48	9.56 ± 4.95	0.280 (0.074)
Epilepsy type^d^^,^^e^				
Focal, *n* (%)	17 (33.33)	10 (40)	21 (30)	0.656 (0.076)
Generalized, *n* (%)	34 (66.67)	15 (60)	49 (70)	
MRI^d^^,^^e^				
Normal, *n* (%)	17 (45.95)	4 (19.05)	24 (42.86)	0.101 (0.201)
Abnormal, *n* (%)	20 (54.05)	17 (80.95)	32 (57.14)	
Anti-seizure medications^d^				
Monotherapy, *n* (%)	38 (70.37)	0 (0)	46 (64.79)	<0.001 (0.507)
Combination therapy, *n* (%)	16 (29.63)	25 (100)	25 (35.21)	
Having Developmental delay^d^				
Yes, *n* (%)	17 (31.48)	17 (68)	23 (32.39)	0.003 (0.277)
No, *n* (%)	37 (68.52)	8 (32)	48 (67.61)	

CE, controlled epilepsy; DRE, drug-resistant epilepsy; HC, healthy control; NDRE, non-drug-resistant epilepsy; NHS3, National Hospital Seizure Severity Scale; SD, standard deviation.

a Data are presented as mean ± SD.

b Kruskal–Wallis test.

c NHS3 score of the worst seizure for those at the age of 6 years and older.

d Pearson Chi-square test.

e Valid percent. *Significant at *P*-value <0.05.

f effect size: *f* for Kruskal–Wallis test with threshold of 0.1, 0.25 and 0.4 for small, medium, and large effects, respectively; *ω* for chi-square test with thresholds of 0.1, 0.3 and 0.5 for small, medium and large effects, respectively.

### Metabolomics profiling of the study population

The LC-MS metabolomics profile identified 16,450 mass ion features, with 10,138 in positive mode and 6,312 in negative mode. A 50% frequency filter applied across at least one condition reduced the number of features to 9,036 for statistical analysis. Two comparisons were conducted: the epilepsy profile (CE vs. HC) and the drug-resistant epilepsy profile (DRE vs. NDRE).

### Metabolic profile of epilepsy panel (comparing CE to HC)

The OPLS-DA model demonstrated a clear separation between the CE and HC groups (Figure 1A), with R2Y: 0.976 and Q2: 0.774, indicating good model fit and predictive ability. A volcano plot (unpaired t-test, FDR-adjusted *p*-value < 0.05, FC 2) was used to compare the CE and HC groups (Figure 1B). Of 9,036 features, 1,287 metabolites showed significant dysregulation: 623 were upregulated, and 664 were downregulated in CE compared to HC. Among the 1,287 significantly altered LC–MS features identified in the CE-versus-HC comparison, 219 were putatively annotated as human endogenous metabolites and had identifiers compatible with the pathway-analysis database. Therefore, only these 219 metabolites were included in pathway analysis; unannotated, ambiguously annotated, exogenous, and unmappable features were excluded. After matching human endogenous metabolites in HMDB, we selected 219 endogenous metabolites for pathway analysis, with 75 increased and 144 decreased in CE relative to HC. The most affected metabolites are illustrated in the heatmaps (Figure 1C for upregulated and Figure 1D for downregulated). Pathway analysis (Figure 1E) revealed that several metabolite groups were significantly affected in CE, including pyrimidine and purine metabolism, phenylalanine, tyrosine, and tryptophan biosynthesis, pyruvate metabolism, and glycolysis/gluconeogenesis. Notably, metabolites involved in fatty acid *β*-oxidation, glycerophospholipids, and ganglioside metabolism were also significantly altered in CE versus HC, despite these pathways not appearing in the pathway analysis.

**Figure 1:**
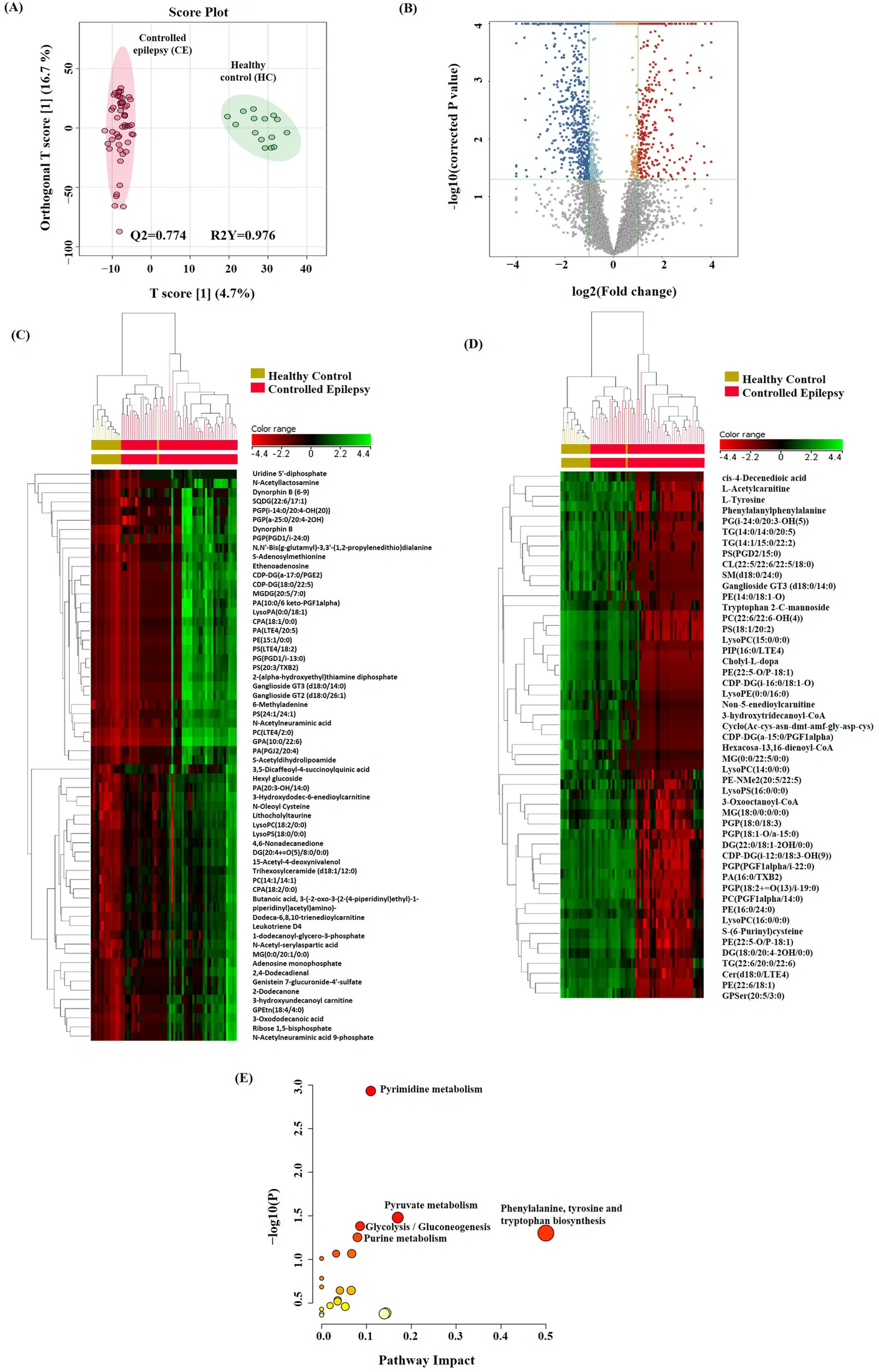
Epilepsy profile. **(A)** OPLS-DA model shows clear separation between the controlled epilepsy (CE) and the healthy control groups (HC). The robustness of the model was evaluated using its fitness (R2Y = 0.976) and predictive ability (Q2 = 0.774) on a larger dataset (*n* = 100). **(B)** Volcano plot shows epilepsy panel, comparing CE and HC groups, based on (*n* *=* 9,036) (unpaired t-test, FDR-adjusted *p*-value < 0.05, FC 2). A group of 1,287 dysregulated metabolites was identified, where 623 and 664 metabolites were up-(red) and down-regulated (blue) between the two groups, respectively. **(C,D)** Heat map of the top altered metabolites, based on endogenous metabolites (*n* = 219) between CE and HC, upregulated or down-regulated, respectively. The higher values (green) indicate higher metabolite abundance, and the lower values (red) indicate lower abundance. **(E)** Pathway analysis based on endogenous metabolites (*n* = 219) between CE and HC.

Pathway analysis was restricted to statistically significant features putatively annotated as human endogenous metabolites and mapped to identifiers recognized by the pathway-analysis database. Features with uncertain or ambiguous annotations, exogenous compounds, and features without compatible pathway identifiers were excluded. Metabolites involved in pyruvate metabolism and the glycolysis/gluconeogenesis pathway, such as HE-TDP and S-acetyldihydrolipoamide intermediates of the pyruvate dehydrogenase complex (PDHc), were significantly higher in the CE group than in HC. Disruption of pyrimidine metabolism led to a notable buildup of intermediates, including deoxyuridine, uridine 5'-diphosphate (dUDP), and deoxythymidine diphosphate (dTDP), in the CE group. Regarding purine metabolism, CE individuals showed significantly increased levels of the DNA damage marker ethenoadenosine and the DNA methylation product 6-Methyladenine compared with HC. Additionally, adenosine monophosphate (AMP) levels were markedly higher in CE. Moreover, a disturbance in the biosynthesis of phenylalanine, tyrosine, and tryptophan was indicated by significantly lower levels of these amino acids, as well as the phenylalanine-phenylalanine dipeptide.

Dysregulation of fatty acid *β*-oxidation was observed in CE, with notably higher levels of several medium- and long-chain fatty acid intermediates and fatty acylcarnitine conjugates, such as -Oxododecanoic acid and 3-hydroxyundecanoyl-carnitine, compared to HC. Conversely, this disruption also led to decreased synthesis of several fatty acyl-CoA intermediates, which were significantly lower in CE than in HC, including 13Z, 16Z-docosadienoyl-CoA and undeca−6,9-dienoyl-CoA. Additionally, levels of L-acetylcarnitine and pantetheine were reduced.

Several glycerophospholipids, including derivatives of cytidine diphosphate-diacylglycerol (CDP-DG), phosphatidic acid (PA), phosphatidylcholine (PC), phosphatidylserine (PS), phosphatidylethanolamine (PE), phosphatidylglycerophosphate (PGP), and phosphatidylglycerol (PG), were significantly elevated in the CE panel. Conversely, levels of other derivatives of these metabolites were lower in CE than in HC. Notably, phosphatidylinositol 4,5-bisphosphate (PIP2), a precursor to key second messengers such as inositol 1,4,5-trisphosphate (IP₃) and diacylglycerol (DAG), was significantly higher in CE patients than in HC.

CE showed a markedly higher level of gangliosides GT2 and GT3 compared to HC, while levels of gangliosides GM3 and GD3 were lower. Additionally, CE exhibited elevated SAM levels.

### Metabolic profile of drug-resistant epilepsy panel (comparing DRE and NDRE)

In the context of the drug-resistant epilepsy panel within the uncontrolled epilepsy group, the comparison of DRE and NDRE using an OPLS-DA model showed reasonable separation between the two groups (Figure 2A), with fitness and predictive metrics of R2Y = 0.956 and Q2 = 0.407. A volcano plot (unpaired t-test with FDR-adjusted *p*-value <0.05 and FC > 2) was used to compare the groups (Figure 2B). Out of 9,036 features, 150 metabolites exhibited significant dysregulation, with 32 upregulated and 118 downregulated in DRE compared to NDRE. Only 26 of these were identified as human endogenous metabolites via HMDB and used for pathway analysis. Among them, 1 metabolite was upregulated and 25 downregulated in DRE versus NDRE. Cholylmethionine was the only metabolite with significantly higher levels in DRE. The heatmap (Figure 2C) visualizes the most altered metabolites. Pathway analysis (Figure 2D) revealed significant disruptions in pyruvate metabolism, glycolysis/gluconeogenesis, and the TCA cycle in DRE, along with some alterations in cysteine and methionine metabolism. Notably, metabolites involved in glycerophospholipid and ganglioside metabolism were also significantly affected in DRE compared to NDRE.

**Figure 2:**
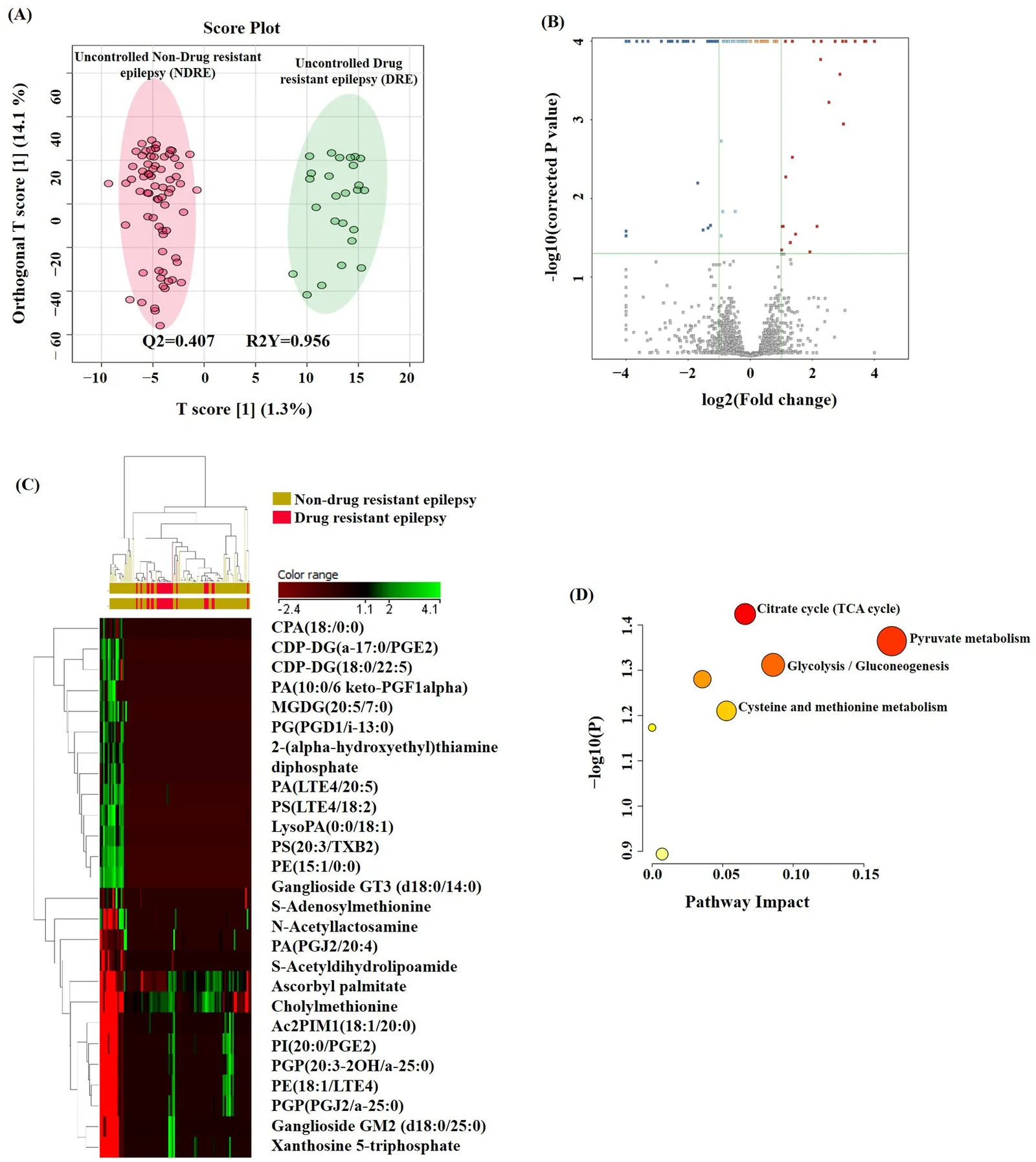
Drug resistance epilepsy profile. **(A)** OPLS-DA shows clear separation between two groups of uncontrolled epilepsy (UCE) patients: drug-resistant epilepsy (DRE) and non-drug-resistant epilepsy (NDRE). The robustness of the model was evaluated by its fitness (R2Y = 0.956) and predictive ability (Q2 = 0.407) in a larger dataset (*n* = 100). **(B)** The volcano plot displays the drug-resistant epilepsy panel, comparing two groups of UCE patients: DRE and NDRE (*n* = 9,036), based on an unpaired t-test (FDR-adjusted *p*-value < 0.05, FC 2). A total of 150 dysregulated metabolites were identified, with 32 upregulated (red) and 118 downregulated (blue) between the two groups. **(C)** Heat map analysis highlights the top altered endogenous metabolites (*n* = 26) between drug-resistant epilepsy (DRE) and non-drug-resistant epilepsy (NDRE) groups. **(D)** Pathway analysis based on endogenous metabolites (*n* = 26) comparing *P* versus T.

Unlike the epilepsy panel, levels of HE-TDP and S-Acetyldihydrolipoamide intermediates were notably lower in DRE compared to uncontrolled NDRE. Additionally, patients with DRE had significantly lower SAM levels than those with NDRE.

Our findings showed a distinct change in glycerophospholipids, with all intermediates and products of the phospholipid biosynthetic pathway significantly reduced in DRE patients compared with uncontrolled NDRE. This included lysoPA, PA, CDP-DG, and key phospholipids such as phosphatidylinositol (PI), PS, and cardiolipin precursors like PG and PGP. The CDP-ethanolamine branch of the Kennedy pathway was also downregulated, with PE levels notably lower in DRE than in NDRE. However, unlike in the epilepsy panel, PIP and PIP2 were not identified as altered metabolites in the DRE panel relative to NDRE. Regarding gangliosides, we showed that GM2 and GT3 levels were significantly lower in DRE than in uncontrolled NDRE patients.

## Discussion

Our study revealed impairments in several energy production pathways in pediatric patients with controlled epilepsy compared to healthy controls. These include the glycolysis/gluconeogenesis pathways, the pyruvate pathway, and metabolites related to fatty acid *β*-oxidation, glycerophospholipid, and ganglioside metabolism. The disruption of these energy pathways could be postulated to result from oxidative stress and mitochondrial dysfunction associated with epilepsy, which could lead to low ATP production, a reduced seizure threshold, and excessive neuronal firing. This energy deficit and mitochondrial oxidative stress increase the production of reactive oxygen species (ROS) (27). ROS further damage lipids, proteins, and nucleic acids, while depleting antioxidant mechanisms such as glutathione (27). This creates a vicious cycle of increasing ROS production and mitochondrial dysfunction, eventually contributing to the development and progression of chronic epilepsy (27, 28). In drug-resistant epilepsy (DRE), besides disruptions in pyruvate and glycolysis pathways observed in this study, the TCA cycle and the antioxidant pathway involving cysteine and methionine metabolism were also significantly affected. Additionally, several metabolite groups were altered, including glycerophospholipids essential for mitochondrial function and the electron transport chain, as well as numerous gangliosides critical for mitochondrial and neuronal health. In this section, we discuss how the findings of this metabolomics study that support the proposed hypothesis of the involvement of mitochondrial dysfunction and oxidative stress in the pathogenesis of epilepsy and DRE. Functional assays of mitochondrial function should be addressed in future experiments. Schematic overviews of the altered pathways proposed to contribute to the pathophysiology of epilepsy and DRE are presented in Figures 3, 4, respectively.

**Figure 3:**
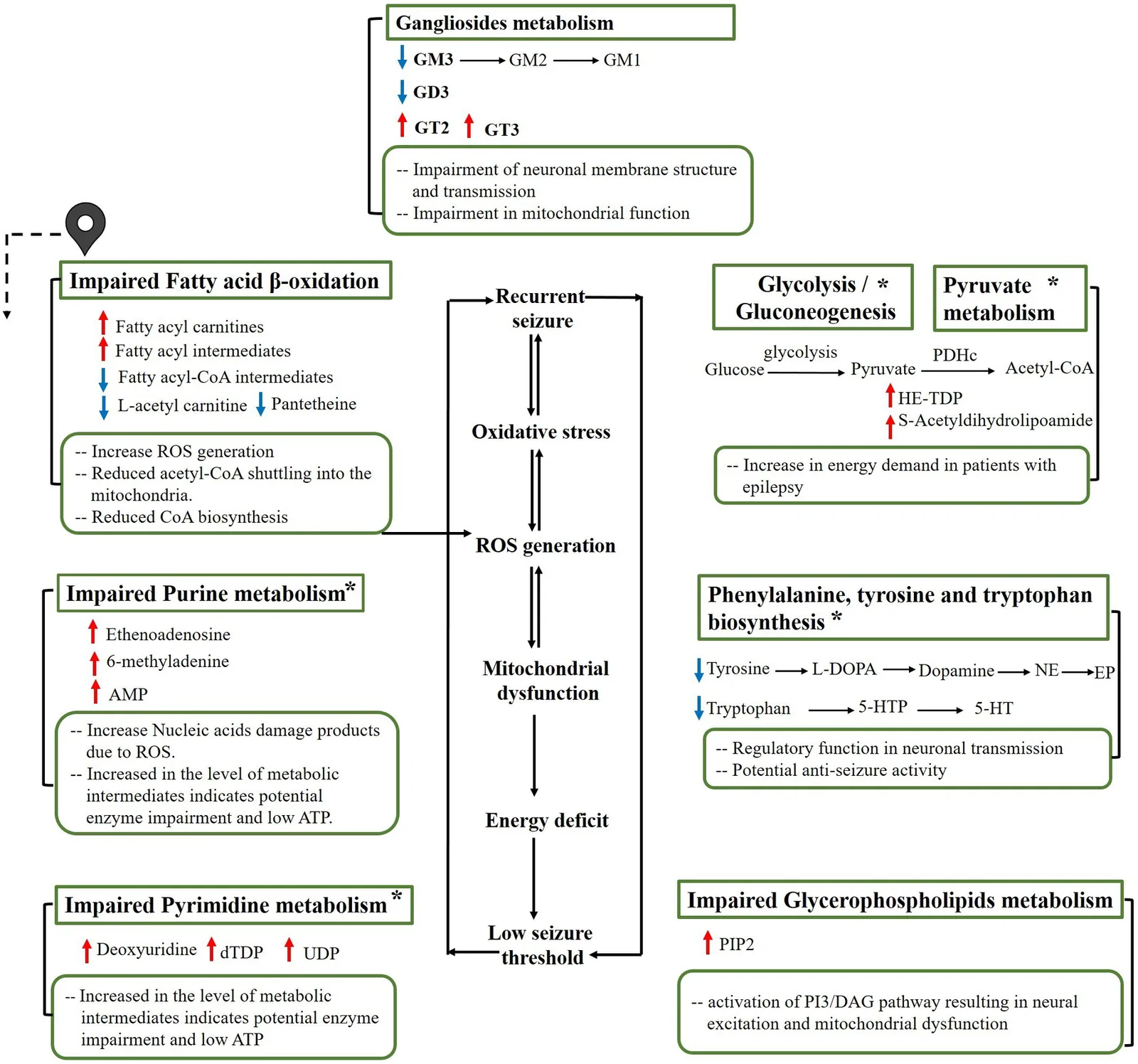
The altered energy metabolism pathways and related metabolites in pediatric epilepsy patients. Potential links to oxidative stress, reactive oxygen species (ROS) production, and mitochondrial dysfunction are highlighted here. * denotes pathways with significant changes. Red arrows indicate metabolites that are significantly increased, while blue arrows indicate metabolites that are decreased in epilepsy compared to healthy controls. Key metabolites include AMP, XTP, dTDP, UDP, PIP2, NE, EP, 5-HTP, 5-HT, PDHc, HE-TDP, and GM.

**Figure 4:**
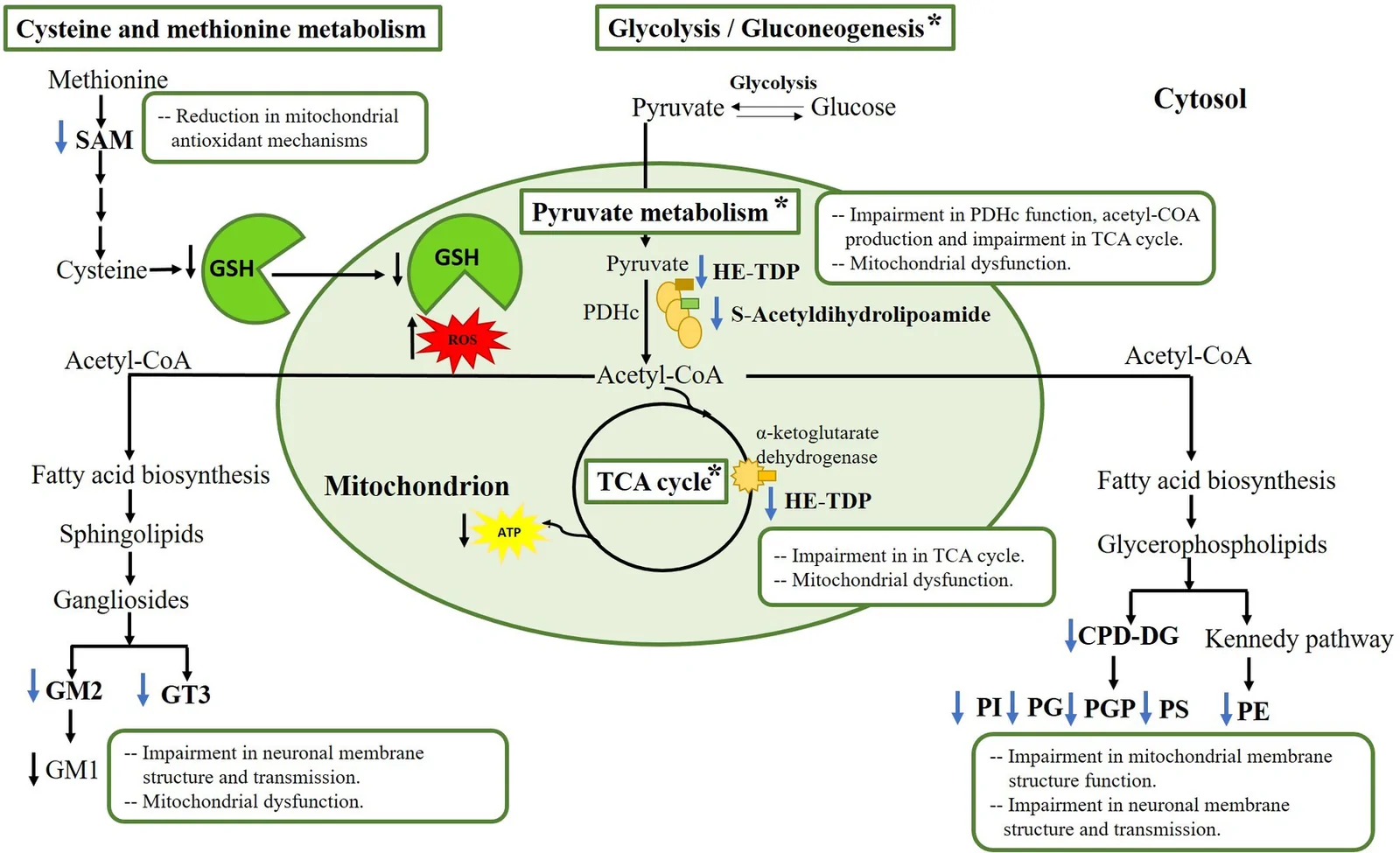
The altered energetic metabolic pathways and the associated metabolites in pediatric patients with DRE compared to those with NDRE. These changes potentially indicate mitochondrial dysfunction, evidenced by significant disruptions in glycolysis/gluconeogenesis, the pyruvate pathway, and the TCA cycle, along with alterations in cysteine and methionine metabolism. An asterisk (*) denotes pathways that are significantly affected. Blue arrows highlight metabolites that are significantly decreased in the DRE group relative to NDRE. Abbreviations include: PDHc for pyruvate dehydrogenase complex; HE-TDP for 2-(alpha-hydroxyethyl) thiamine diphosphate; TCA for tricarboxylic acid cycle (Krebs cycle); GM for gangliosides; SAM for S-adenosylmethionine; ROS for reactive oxygen species; GSH for glutathione; CPD-DG for cytidine diphosphate-diacylglycerol; PI for phosphatidylinositol; PG for phosphatidylglycerol; PGP for phosphatidylglycerophosphate; and phosphatidylserine, PE for phosphatidylethanolamine.

We demonstrated an alteration in fatty acid *β*-oxidation in CE (Figure 4). Compared to HC, several medium- and long-chain fatty acid intermediates and fatty acylcarnitine conjugates were significantly elevated in CE. Conversely, the levels of several fatty acyl-CoA intermediates were notably reduced in CE. Fatty acid *β*-oxidation is one of the primary energy production pathways in mitochondria (29). Its end product, acetyl-CoA, enters the TCA cycle to generate ATP (29). Here, significantly higher levels of carnitine conjugates of fatty acids were observed in CE compared to HC. Despite the essential role of the shuttle system that transports acyl groups via carnitine, the accumulation of carnitine conjugates indicates a disruption of fatty acid *β*-oxidation in mitochondria and suggests mitochondrial dysfunction (30). Furthermore, the build-up of fatty acyl intermediates is thought to be a major factor in ROS generation, leading to a vicious metabolic cycle that causes further damage, mitochondrial dysfunction, and a state of energy deficiency (31) (Figure 3). While this accumulation might be genetically mediated through the presence of impaired metabolizing enzymes (32), it can also arise from mitochondrial dysfunction, such as the oxidative stress observed in epilepsy (3). A previous study using a Kainic acid model of temporal lobe epilepsy in rats supported our findings, showing that acylcarnitines were upregulated in the hippocampus during the acute and latent seizure phases and in plasma during the acute phase (33). The increased levels of acylcarnitines could indicate an enhanced utilization of fatty acids as an alternative energy source, as suggested earlier (33). Unlike earlier reports that showed reduced free fatty acids in adults with epilepsy, attributed to increased utilization during seizures (13), our results point instead to elevated levels of carnitine and CoA fatty acid conjugates, suggesting impaired *β*-oxidation.

Interestingly, the controlled epilepsy panel showed a significant decrease in L-acetylcarnitine levels. This molecule transports acetyl groups between the cytosol and the mitochondrial membrane (30). The reduced L-acetylcarnitine may reflect lower acetyl-CoA levels, suggesting decreased production due to impaired fatty acid *β*-oxidation. Additionally, we observed a notably lower level of pantetheine in CE than in HC. Since pantetheine is necessary for CoA biosynthesis (34), its deficiency leads to reduced C3A levels, indicating mitochondrial dysfunction and disrupted fatty acid oxidation, where CoA is vital for enzyme activity (29). To our knowledge, this is the first metabolomics study to report significant impairments in fatty acid *β*-oxidation metabolites in pediatric epilepsy patients (Figure 3).

Our study revealed alterations in glycolysis/gluconeogenesis and pyruvate metabolism in patients with epilepsy. Pyruvate metabolism via PDHc is a critical process in energy metabolism (35). In the mitochondria, PDHc converts pyruvate, the product of glycolysis, into acetyl-CoA (Figure 3), the substrate for both the TCA cycle in the mitochondria and fatty acid biosynthesis in the cytosol (Figure 4) (35). Impairment of PDHc function can result in TCA cycle dysfunction, leading to mitochondrial dysfunction and an energy deficit (3, 27). Congenital deficiency of PDHc is associated with variable frequency of epilepsy (36), and reduced enzymatic activity of PDHc was demonstrated in an acute murine seizure model (37). The action of PDHc involves, in its first step, pyruvate decarboxylation utilizing thiamine pyrophosphate (TPP) [also called thiamine diphosphate (TDP), vitamin B1] as a cofactor. The resulting hydroxyethyl-TDP then reacts with lipoamide to produce S-acetyldihydrolipoamide and regenerate TDP (35). In this study,significantly higher levels of HE-TDP and S-acetyldihydrolipoamide were observed in patients with CE compared with HC. This could be attributed to increased energy demand in patients with epilepsy. However, their levels were significantly lower in DRE than in NDRE. This indicates selective depletion of these essential components of the PDHc reaction in DRE patients, making them, particularly S-acetyldihydrolipoamide, potential biomarkers for the diagnosis and identification of DRE. In addition, these metabolites can be postulated to be potential therapeutic targets for DRE. In animal models, thiamine supplementation has been demonstrated to have anticonvulsant activity (6, 38). This might suggest that supplementation with vitamin B1 (thiamine pyrophosphate) could rescue the alteration in pyruvate metabolism observed in patients with DRE. To our knowledge, this study is the first metabolic report to show impairment of the pyruvate pathway in humans with DRE and to elucidate the mechanism of this impairment, providing two potential biomarkers and proposed therapeutic targets for DRE: S-acetyldihydrolipoamide and HE-TDP. However, future clinical studies are required to confirm the therapeutic potential of these metabolites in epilepsy patients.

The above findings were further supported by a significant change in the TCA cycle, as shown in the DRE panel but not in the CE panel. Likewise, TCA pathway intermediates were reduced in pediatric epilepsy patients. The decreased HE-TDP levels observed in DRE could disrupt the TCA cycle through two mechanisms: either by impairing the activity of *α*-ketoglutarate dehydrogenase, a key enzyme that requires TDP as a cofactor (39) or by limiting acetyl-CoA input, as previously described (Figure 4). Importantly, this study is the first to establish a metabolic link between HE-TDP reduction and TCA cycle impairment in humans with DRE.

The pyruvate pathway is involved in the upstream steps of fatty acid and glycerophospholipid biosynthesis (40). As discussed earlier, changes in the pyruvate pathway could explain the altered glycerophospholipid levels observed in our study. In this research, the phospholipid pathway in the CE panel did not show a clear pattern, with several glycerophospholipid derivatives either higher or lower in the CE group than in HC. However, in DRE, the levels of all intermediates and products of the CDP-DG and Kennedy pathways for phospholipid biosynthesis, detected in this study, were consistently lower in the DRE panel compared to uncontrolled NDRE. Additionally, the levels of all intermediates and products of the phospholipid biosynthesis pathway, such as PA, lysoPA, PI, PG, PGP, PS, and PE, were significantly reduced in DRE patients compared to uncontrolled NDRE (Figure 4). Consistent with our findings, brain tissue samples from patients undergoing surgery for medically intractable epilepsy showed decreased levels of PE and PC in high-spiking brain tissue compared to low-spiking tissue (31). Conversely, in non-drug-resistant epilepsy, reduced levels of fatty acids and glycerophospholipids, including PI, PE, and PC, were observed in the sera of pediatric patients using metabolomics and lipidomic analysis (4). Lai W et al. reviewed several studies in animal models of epilepsy that reported alterations in various glycerophospholipids, including lysoPC, PC, PA, LysoPA, and PS (6). The studies varied in the model used, sample type, and analytical platform employed (6). This makes our study the first metabolomics study to consistently demonstrate low levels of glycerophospholipids in the sera of pediatric patients with DRE.

Glycerophospholipids such as PE and PS are crucial for maintaining membrane structure, neuronal transmission, neurotransmitter release, and mitochondrial function (Figure 4). In this study, DRE was associated with significantly reduced levels of PS and PE. PS is the primary precursor for mitochondrial PE, produced via the *in situ* decarboxylation of PS by the mitochondrial enzyme phosphatidylserine decarboxylase. Thus, the novel finding of low PS levels in this study may suggest a role in the development of drug-resistant epilepsy in children, potentially through impaired mitochondrial function caused by decreased PE biosynthesis. Animal models of epilepsy also show reductions in PS and PE levels (6). Dysregulated PS metabolism has been linked to various CNS diseases, and supplementation has been shown to benefit conditions such as Parkinson's disease, major depressive disorder, and stroke (41). Similarly, we propose here that PS supplementation combined with traditional ASMs could have therapeutic potential for epilepsy, but this warrants further clinical research.

Regarding PI and its derivatives, we observed significantly higher PIP2 levels in CE patients than in healthy controls (HC), whereas PIP2 levels did not differ between the DRE and NDRE groups. PIP2 serves as a precursor in IP3/DAG signaling and is hydrolyzed by phospholipase C (PLC) to generate the second messengers IP₃ and DAG. These messengers regulate cellular processes that influence seizure activity, ultimately increasing neuronal excitability (see Figure 4). Previous studies have linked IP3/DAG signaling to focal epilepsy. Additionally, calcium overload triggered by IP3 may cause mitochondrial dysfunction and impair ATP production, leading to neuronal impairments. A recent study also showed neuroprotective effects through PLC-*β* inhibition in animal models (42). Supporting our findings of altered PI levels in epilepsy, earlier research reported elevated PI levels in the dorsal hippocampus one hour after an acute epileptic seizure in mice compared to controls (43). To our knowledge, this is the first metabolomics study to show changes in PI, PIP, and PIP2 levels in the serum of pediatric epilepsy patients, independent of seizure episodes.

Gangliosides are glycosphingolipids with a ceramide backbone linked to one or more sialic acid residues, forming mono-sialylated (GM), di-sialylated (GD), or tri-sialylated (GT) gangliosides. Sphingolipid biosynthesis depends on fatty-acyl CoA, ultimately derived from pyruvate via acetyl-CoA (40). As shown in this study, disruption of PDHc function impairs sphingolipid biosynthesis, leading to the ganglioside level changes observed here (Figure 4). The brain's most abundant gangliosides are GM1 (17%), GD1a (26%), GD1b (27%), and GT1b (24%), while GM3 and GM2 each account for less than 2% (44). Of these, GM1 is the most extensively studied due to its protective role in the brain (44, 45). Despite its low abundance, GM3 is essential in the ganglioside biosynthetic pathway, serving as the common precursor for all other gangliosides, including GM1 (44). GM3 is converted to GM2, which then produces GM1 (44). In our study, reduced GM3 in CE and GM2 in DRE compared with HC and uncontrolled NDRE suggest potential GM1 deficiency. Previous research indicates that deficiencies in GM3 synthase and GM2 synthase result in decreased GM3, GM2, and downstream derivatives such as GM1, possibly triggering epileptic seizures (45). Studies have reported the absence of GM3 and its derivatives in the plasma of patients with infantile-onset symptomatic epilepsy, with reduced GM1 and GD1a levels observed in the CSF of patients with West syndrome. Both deficits and excesses of gangliosides are linked to neurodegenerative disorders, even with subtle level changes or altered expression (45). Thus, the expression changes we observed in the epilepsy panel could be clinically significant. Gangliosides also play roles in mitochondrial function (46). Case reports of early-onset refractory epilepsy show mitochondrial dysfunction associated with GM3 synthase deficiency and the absence of GM3 and its derivatives (46). Moreover, GM1 has been shown to regulate mitochondrial function (47). Consequently, the low levels of GM3 in CE and GM2 in DRE may relate to decreased GM1, which is vital for neuronal membrane integrity and mitochondrial function (45), in line with Figures 3, 4. This supports our hypothesis of impaired mitochondrial function and energy production in DRE, potentially mediated by PDHc impairment. A rat model of temporal lobe epilepsy revealed downregulation of gangliosides in the hippocampus and plasma at various time points (33). A recent metabolomics study highlighted the involvement of sphingolipid metabolism and a decrease in sphingosine 1-phosphate in the serum of adults with DRE (14). Our study is the first pediatric metabolomics study to show clear reductions in serum gangliosides, specifically GM3 and GM2, in patients with epilepsy or DRE, respectively. Based on these findings we propose that ganglioside supplementation might have therapeutic potential for epilepsy or DRE which warrants future investigation. Supporting this, a recent mouse study demonstrated that dietary supplementation with GM3 and GD3 improved cognitive function and neurogenesis in the hippocampus of GM3 synthase-deficient mice (48).

Impaired methylation capacity and elevated homocysteine levels were identified as key biochemical features of epilepsy (49). Our research indicates that the cysteine-methionine metabolic pathway is disrupted in DRE, correlating with markedly lower SAM levels in DRE patients compared with NDRE, whereas CE patients show higher SAM levels than HC. SAM, the primary methyl donor in cells, initiates the methionine/cysteine pathway (50). Cysteine, a critical precursor for glutathione synthesis, an antioxidant vital for mitochondrial defence against respiratory oxidative stress (51), may decline when SAM is depleted, reducing glutathione production and impairing mitochondrial antioxidant function postulated in this study. This can disrupt the TCA cycle, which is sensitive to oxidative stress (52) (see Figure 4). Because SAM biosynthesis depends on ATP, the hypothesized mitochondrial dysfunction and reduced energy production can impair methionine adenosyltransferase (MAT), the enzyme that converts methionine to SAM (50). Supporting this, animal studies show that SAM supplementation has anticonvulsant, memory-enhancing, and antioxidant effects in a rat model of pentylenetetrazole-induced epilepsy (53). To our knowledge, this is the first metabolomics study to identify significantly low SAM levels in DRE patients, proposing SAM as a potential biomarker and therapeutic target for DRE.

Mitochondrial dysfunction, oxidative stress, and ROS generated during seizures in patients with epilepsy can directly damage mitochondrial DNA and proteins, including enzymes involved in purine and pyrimidine metabolism (3). These enzymes may include those critical for nucleotide processing. Additionally, the suggested mitochondrial dysfunction can impair purine and pyrimidine biosynthesis by reducing ATP production (see Figure 3). In our study, we found significantly higher levels of several intermediates in the purine and pyrimidine pathways in CE compared to HC. Damage to thymidylate kinase, which converts deoxythymidine monophosphate (dTMP) to dTDP, might lead to the accumulation of early dTMP precursors such as deoxyuridine and UDP. Moreover, damage to nucleoside diphosphate kinase could hinder the conversion of dTDP to dTTP, causing dTDP buildup, as shown (see Figure 3).

Remarkably, our study shows that alterations in purine metabolism are associated with elevated levels of the direct DNA damage product ethenoadenosine in CE compared with HC. PS is a damaged analogue of riboATP and is considered a marker of nucleic acid damage (54). Therefore, the high level of ethenoadenosine strongly suggests potential mitochondrial DNA damage in epilepsy patients (Figure 3). Interestingly, we also demonstrated significantly higher levels of 6-Methyladenine in patients with CE compared with HC. Notably, the ribose derivative of 6-methyladenine, 6-methyladenosine, can be induced by oxidative stress and DNA damage (55). Recently, 6-Methyladenine itself was found to be widely present in the human genome, and its low levels in DNA promote tumorigenesis (56), suggesting a possible role in DNA repair. Therefore, we suggest that the elevated levels of 6-Methyladenine in the epilepsy panel indicate ongoing DNA damage and repair. However, in line with our hypothesis, mitochondrial DNA damage resulting from oxidative stress was associated with epileptogenesis in a murine model (57). A high level of AMP was also observed in this study, suggesting a potential blockage in the conversion of AMP to ATP. Alterations in purine metabolism in epilepsy have been reported previously; however, in an acute animal model of epilepsy, reduced levels of AMP, ADP, and ATP were observed in the piriform cortex during the acute phase of seizure (58).

Phenylalanine, tyrosine, and tryptophan biosynthetic pathways were also significantly altered in the epilepsy panel compared with HC. This pathway produces the neurotransmitters dopamine, norepinephrine, epinephrine, and serotonin (59). These neurotransmitters regulate epilepsy and reduce seizure susceptibility (59, 60) (Figure 3). Therefore, disruption of this pathway impairs epilepsy regulation. Consistent with our findings, previous reports in both adults and pediatric patients with epilepsy have shown reduced L-tryptophan levels.

However, several key limitations must be noted. First, the study involved only pediatric patients with epilepsy, and it is well established that the metabolic profile varies with age (61). Thus, the current findings would not be expected to generalize to the population of adults with epilepsy. In addition, we could not account for the effects of various medications and differing dietary intake as confounders in these metabolic measures. In addition, because the healthy control group consisted of patients drawn for routine blood work during clinical visits, it was relatively small (15 patients) compared with the group of children with epilepsy classified as having CE (54 patients). Thus, this study had reduced statistical power and was subject to several biases, including increased sensitivity to outliers and unequal variance between groups. In addition, the metabolomic differences found between healthy children and children with CE must be interpreted with caution. In addition to these limitations, patients in the NDRE group are clinically heterogeneous. These individuals have seizures that are still uncontrolled with treatment; however, their seizures are not resistant to all anti-seizure medications (ASMs). Depending on response to ASMs, some individuals in this group will be classified as having DRE, whereas others will be classified as having CE. Thus, increased within-group metabolic variability in NDRE patients may reduce specificity when comparing metabolic profiles from the NDRE group with those from patients with DRE. Furthermore, the proposed involvement of mitochondrial dysfunction and oxidative stress was inferred from pathway-associated metabolite alterations and was not confirmed by direct functional, enzymatic, mitochondrial respiration, or oxidative-stress measurements. In addition, a major limitation of the current untargeted approach used in this study is the level of annotation of the identified metabolites. Although eight of the various metabolites identified in this study were confirmed using authenticated reference standards for the identified compounds (MSI level 1) (17), putative identification was performed for the remaining metabolites using accurate mass, as well as information regarding isotopic distribution, as well as information regarding fragmentation pattern using MS/MS data (MSI level 2) (17). Thus, the proposed biomarkers as well as putatively identified metabolites that may have some therapeutic relevance, ie, some metabolite that may be used as a probe for some aspect of seizure induction or some antiepileptic action, would remain to be confirmed using appropriate reference standards under the appropriate analytical conditions, quantitative analysis using targeted methodology, as well as validation studies involving independent patient samples.

## Conclusion

This study further explores the pathophysiological mechanisms of epilepsy, highlighting impairments in multiple energetic pathways hypothetically linked to oxidative stress. In the epilepsy group, these alterations were associated with disruptions in pyrimidine and purine metabolism, leading to nucleic acid degradation, as evidenced by high levels of metabolites such as ethenoadenosine and 6-Methyladenine. We demonstrate, for the first time, significantly decreased levels of HE-TDP (the active form of vitamin B1), S-Acetyldihydrolipoamide, SAM, and GM2 ganglioside in the DRE group, which could serve as potential diagnostic biomarkers. However, these markers require validation using more sensitive techniques, such as targeted LC-MS, and in larger patient cohorts. Interestingly, we postulate that HE-TDP, SAM, GM2, and mitochondrial glycerophospholipids (PS and PE) may also serve as therapeutic targets in DRE. Therefore, we hypothesize that supplementation with vitamin B1, SAM, GM2, PS, and PE might be a promising strategy to enhance energetic pathways and mitochondrial and neuronal functions. Further research is necessary to assess the clinical viability of these supplementation approaches in DRE.

## Data Availability

The original contributions presented in the study are included in the article/Supplementary Material, further inquiries can be directed to the corresponding authors.
